# Blackcurrant (*Ribes nigrum*) Extract Prevents Dyslipidemia and Hepatic Steatosis in Ovariectomized Rats

**DOI:** 10.3390/nu12051541

**Published:** 2020-05-25

**Authors:** Naoki Nanashima, Kayo Horie, Kanako Yamanouchi, Toshiko Tomisawa, Maiko Kitajima, Indrawati Oey, Hayato Maeda

**Affiliations:** 1 Department of Bioscience and Laboratory Medicine, Hirosaki University Graduate School of Health Sciences, 66-1 Hon-cho, Hirosaki, Aomori 036-8564, Japan; k-horie@hirosaki-u.ac.jp (K.H.); kanako.8@hirosaki-u.ac.jp (K.Y.); 2 Department of Nursing Sciences, Hirosaki University Graduate School of Health Sciences, 66-1 Hon-cho, Hirosaki, Aomori 036-8564, Japan; tmtott@hirosaki-u.ac.jp (T.T.); kitajima@hirosaki-u.ac.jp (M.K.); 3 Department of Food Science, University of Otago, PO Box 56, Dunedin 9054, New Zealand; indrawati.oey@otago.ac.nz; 4 Riddet Institute, Private Bag 11 222, Palmerston North 4442, New Zealand; 5 Faculty of Agriculture and Life Science, Hirosaki University, 3 Bunkyo-cho, Hirosaki 036-8561, Japan; hayatosp@hirosaki-u.ac.jp

**Keywords:** blackcurrant, dyslipidemia, liver steatosis, ovariectomized, phytoestrogen

## Abstract

Estrogen is involved in lipid metabolism. Menopausal women with low estrogen secretion usually gain weight and develop steatosis associated with abnormal lipid metabolism. A previous study showed that blackcurrant (*Ribes nigrum* L.) extract (BCE) had phytoestrogen activity. In this study, we examined whether BCE improved lipid metabolism abnormalities and reduced liver steatosis in ovariectomized rats, as a menopausal animal model. Twelve-week-old ovariectomized (OVX) rats were fed a regular diet (Ctrl) or a 3% BCE supplemented diet while sham rats were fed a regular diet for three months. Body weight, visceral fat weight, levels of serum triglycerides, total cholesterol, and LDL cholesterol decreased in the BCE-treated OVX and sham rats, but not in OVX Ctrl rats. The results of hematoxylin and eosin staining revealed that BCE decreased the diameters of adipocytes and the nonalcoholic fatty liver disease activity score. Furthermore, quantitative RT-PCR indicated a decreased expression of hepatitis-related genes, such as tumor necrosis factor-α, *IL-6*, and *IL-1β* in OVX rats after BCE treatment. This is the first study that reported improvement of lipid metabolism abnormalities in OVX rats by BCE administration. These results suggest that the intake of BCE alleviated dyslipidemia and prevented nonalcoholic steatohepatitis during menopause in this animal model.

## 1. Introduction

Estrogen is directly related to lipid metabolism. After menopause, estrogen levels suddenly decrease. Previous studies found that menopausal women and mice with decreased estrogen secretion experience an increase in weight and symptoms of menopause, such as abnormal lipid metabolism and hepatic steatosis [1,2,3]. In postmenopausal women, total cholesterol (TC), LDL cholesterol (LDL-C), and triglyceride (TG) contents are increased [4], and these changes promote arteriosclerosis and adversely affect the heart and blood vessels [5,6]. Furthermore, dyslipidemia or hepatic steatosis induces nonalcoholic fatty liver disease (NAFLD) or nonalcoholic steatohepatitis (NASH) [7], leading to diseases that are major threats to public health, such as cirrhosis and hepatocellular carcinoma [8]. Estrogen plays an important role in liver lipid metabolism, and its deficiency increases the risk of NAFLD and NASH with menopausal dyslipidemia [2,9]. Thus, decreased estrogen secretion adversely affects menopausal women.

Phytoestrogens are a chemically diverse group of plant compounds with estrogenic effects in animals. Phytoestrogens, which include isoflavones, lignans, coumestans, flavonoids, and resveratrol, are present in several foods [10,11,12,13]. More importantly, some reports indicated that daily intake of phytoestrogen reduced climacteric symptoms [14]. Recently, we reported that blackcurrant (*Ribes nigrum* L.) extract (BCE) had phytoestrogen activity by signaling through both estrogen receptors α and β [15,16].

Blackcurrant contains high levels of polyphenols, especially four anthocyanins, cyanidin-3-glucoside, cyanidin-3-rutinoside, delphinidin-3-glucoside, and delphinidin-3-rutinoside [17]. These compounds elicited health beneficial effects, such as blood flow improvement and cancer suppression effects. Furthermore, previous studies showed that BCE had a cosmetic effect on the skin [18], alleviated hair loss [19], and improved vascular endothelium function in menopausal model rats [20]. A few studies have reported the effectiveness of BCE in alleviating dyslipidemia and NASH caused by the consumption of a high-fat diet [21,22]. However, there are no reports on whether BCE affects dyslipidemia in menopausal women or animals. Therefore, this study aimed to investigate whether BCE reduced dyslipidemia. Ovariectomized (OVX) rats were used as the menopausal animal model to examine whether BCE was effective in reducing dyslipidemia and associated hepatic steatosis during menopause. This is the first study that reports the effects of BCE treatment on lipid metabolism abnormalities in OVX rats.

## 2. Materials and Methods

### 2.1. Animals and Diets

OVX female Sprague-Dawley and sham surgery rats (12 weeks of age; weight 249.7 ± 10.2 g) were purchased from CLEA Japan Inc. (Tokyo, Japan). The rats were housed in air-conditioned rooms, with a 12 h light/dark cycle and with free access to water and food, at the Institute for Animal Experiments of Hirosaki University Graduate School of Medicine.

The BCE powder, CaNZac-35, was purchased from Koyo Mercantile Co. (Tokyo, Japan). BCE contains high concentrations of polyphenols (37.6 g/100 g BCE) and anthocyanins (38 g/100 g BCE) [16]. Since our previous studies showed that 3% BCE elicited phytoestrogen effects in the skin and vascular endothelium of rats [18,19,20], all rats in this study received an AIN-93M diet, with or without 3% BCE, and were assigned into three groups (n = 9–10 rats/group): 1) OVX rats treated with 3% BCE for 3 months (OVX BCE group), 2) OVX control rats without BCE treatment (OVX Ctrl group), and 3) sham surgery rats without BCE treatment (sham group). Blood, uterus, visceral fat, and liver tissues were collected from euthanized animals after 3 months, and the body, uterus, and liver weights were measured. This experiment was approved by the Animal Research Committee of Hirosaki University (permission number: G16004) and was conducted in accordance with the rules for Animal Experimentation of Hirosaki University.

### 2.2. Biochemical Analysis of Serum

Serum TG, glucose, AST (aspartate transaminase), ALT (alanine transaminase), and γ-GT (γ-glutamyl transferase) levels were examined using SPOTCHEM EZ SP-4430 (ARKRAY, Inc., Kyoto, Japan), while TC, HDL-C, and LDL-C contents were measured using the EnzyChrom HDL and LDL/VLDL Assay Kit (BioAssay Systems, CA, USA). Adiponectin and leptin concentrations were determined using CircuLex Rat Adiponectin ELISA Kit (Circulex, CycLex Co. Ltd., Nagano, Japan) and Rat Leptin ELISA Kit (Yanaihara Institute Co. Ltd., Shizuoka, Japan), respectively.

### 2.3. Histological Analysis of Liver and Adipose Tissues

Each tissue was fixed in 10% formaldehyde and embedded in paraffin for histological examination. Liver and adipose tissue sections (4 μm thick) were mounted onto silane-coated slides. The sections were deparaffinized by passing through xylene and a graded alcohol series before staining with hematoxylin and eosin. Digital images were acquired using a fluorescence microscope (FSX100; Olympus, Tokyo, Japan). Adipocyte diameters were measured, and liver steatosis grades were estimated using NAFLD activity score: steatosis (0–3), lobular inflammation foci (0–2), and hepatocellular ballooning (0–2), quantified according to the criteria proposed by Kleiner et al. [23]

### 2.4. RT-qPCR Analysis

Total RNA was prepared using an RNeasy mini kit (Qiagen ,Valencia, CA, USA) according to the manufacturer’s instructions. RNA was reverse-transcribed into cDNA using PrimeScript RT Master Mix (TaKaRa, Tokyo, Japan). Levels of *TNF-α*, *IL-6,* and *IL-1β* mRNAs were quantified by qPCR using TB Green Premix Ex Taq II (Tli RNaseH Plus; TaKaRa). The PCR amplification protocol consisted of 30 s at 94 °C, 30 s at 58 °C, and 30 s at 72 °C for 40 cycles. Transcript levels were normalized to those of glyceraldehyde 3-phosphate dehydrogenase (GAPDH) cDNA. The primer sequences were as follows (5ʹ→3ʹ) [24]: *TNF-α*, forward ACCACGCTCTTCTGTCTACTG and reverse CTTGGTGGTTTGCTACGAC; *IL-6*, forward TCTCTCCGCAAGAGACTTCCA and reverse ATACTGGTCTGTTGTGGGTGG; *IL-1β*, forward GCAATGGTCGGGACATAGTT and reverse AGACCTGACTTGGCAGAGGA; and *GAPDH*, forward TGAGAACGGGAAGTCTGTCA and reverse TCTCCATGGTGGTGAAGACG. PCR specificity was checked using a melting curve analysis. All samples were analyzed in duplicates, and relative gene expression was calculated according to the 2^-ΔΔ^Ct method [25].

### 2.5. Statistical Analysis

Results are expressed as the mean ± standard deviation. Graphs were generated using the Graph Pad Prism 7.0 ver. 7.03 software (Graph Pad Prism, San Diego, CA, USA). Statistically significant differences were determined using Kruskal–Wallis analysis with the Steel post hoc test using the bell curve for Excel ver. 3.2 software (Social Survey Research Information Co., Ltd., Tokyo, Japan). Results with *p*-values <0.05 were considered statistically significant.

## 3. Results and Discussion

### 3.1. Weight of Body, Visceral Fat, Uterine and Volume of Food Intake

**Figure 1:**
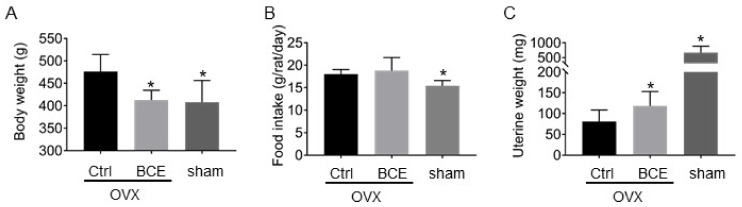
Effect of blackcurrant (*Ribes nigrum* L.) extract (BCE) on (**A**) body weight, (**B**) food intake, and (**C**) uterine weight of rats. Ovariectomized (OVX) rats treated with 3% BCE for 3 months (OVX BCE, n = 9), OVX rats without BCE treatment (OVX Ctrl, n = 10), and sham surgery rats without BCE treatment (sham, n = 9). Data represent the means ± SD. * *p* < 0.05 vs. Ctrl.

Before the experiment, the rats were grown up to 12 weeks old, and there was no significant difference in body weight (data not shown) among the rats. After three months, rats in the OVX Ctrl group increased in body weight compared to those in the sham group. However, BCE intake alleviated weight gain in OVX rats by 14%, comparable to sham rats (15%) (Figure 1A). By examining the food intake, it was 18.9 ± 1.0 g/rat/day in the OVX control group compared to the sham group, but decreased to 15.4 ± 1.2 g/rat/day in the sham group. However, the food intake in the OVX BCE group was 18.9 ± 2.9 g/rat/day, which was not different from that in the OVX control group (Figure 1B). Therefore, in this study, the food intake was the same between OVX control rats and OVX BCE rats. It is known that food intake increases with reduced estrogen levels, but in this research, the food intake of OVX BCE rats was not decreased [26]. Therefore, we concluded that phytoestrogen did not have the same strength as estrogen. It was also suggested that the decrease in the body weights of OVX BCE rats was not due to a decrease in food intake. The amount of BCE employed in the present animal study is equivalent to a daily dose of 1.9 g polyphenols [27], for a 60 kg human. This phenolic intake is considered realistic, and it could be provided by 5.1 g of BCE.

In addition, uterine weight increased with BCE intake (Figure 1C). Estrogens and phytoestrogens enlarge the uterus and promote the thickening of the endometrium. In our previous study, oral administration of 1000 mg/kg BCE to four-week-old young rats for three days without estrogen secretion did not increase the weight of the uterus, but the endometrium was partially thickened. In this study, as BCE was administered for three months, the weight of the uterus may have been affected. Thus, this result confirmed that BCE functioned as a phytoestrogen.

### 3.2. Visceral Adipose Tissue Mass and Adipocyte Sizes

**Figure 2:**
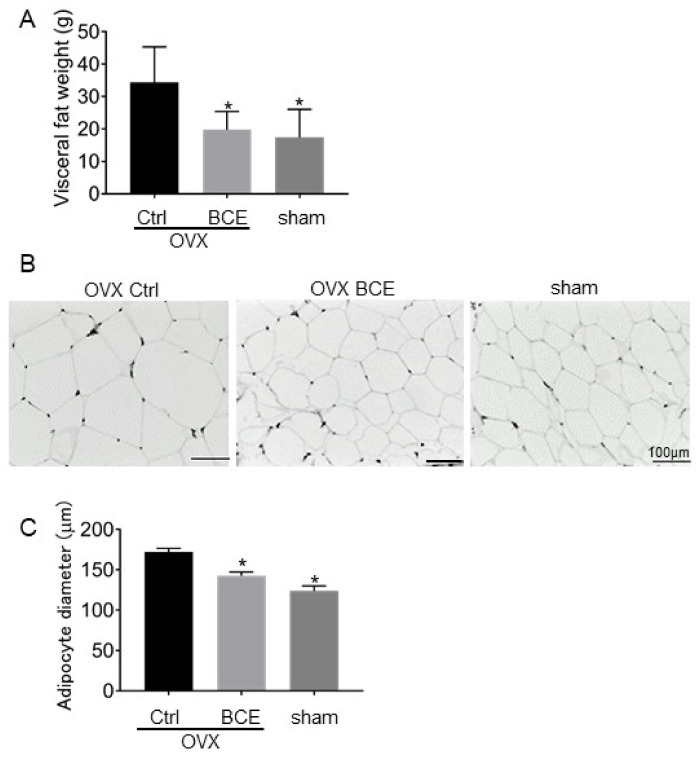
Effect of BCE on visceral adipose tissue mass and size. (**A**) Visceral fat mass, (**B**) images of paraffin-embedded adipocyte hematoxylin and eosin-stained sections of OVX rats treated with 3% BCE for 3 months (OVX BCE, n = 9), OVX rats without BCE treatment (OVX Ctrl, n = 10), and sham surgery rats without BCE treatment (sham, n = 9). Scale bar = 100 μm. (**C**) Average adipocyte diameters were measured in each of the three fields. Data represent the means ± SD. * *p* < 0.05 vs. Ctrl.

OVX rats had a greater visceral adipose tissue mass (34.3 ± 10.9 g) than did sham (17.3 ± 8.8 g) rats (*p* = 0.023). However, OVX BCE rats did not increase in adipose tissue mass (24.3 ± 18.8 g) as much as did OVX Ctrl rats. (Figure 2A). As shown in Figure 2B,C, the average adipocyte diameter also increased in OVX Ctrl rats (172.3 ± 23.1 μm) compared to sham rats (117.2 ± 29.4 μm, *p* < 0.001). However, BCE treatment reduced adipocyte diameters to the levels observed in OVX Ctrl rats (136 ± 24 μm, *p* < 0.001).

Estrogens are known to play an important role in energy control and lipid metabolism, and menopausal women are at an increased risk of lifestyle-related diseases due to their decreased metabolism [28]. Several phytoestrogens have been previously reported to be effective in reducing these risks in OVX rats, but BCE has been shown to have similar effects [29,30].

### 3.3. Serum Lipid Profiles

We investigated whether BCE intake affected serum lipids. TG, TC, and LDL-C levels increased in OVX Ctrl rats compared to sham rats. However, BCE intake reduced these serum lipids. There was no difference in HDL-C (Table 1). These results suggested that abnormal lipid metabolism occurred in OVX Ctrl rats. Serum lipid abnormalities frequently occur in menopausal women and OVX rodents [31,32]. Therefore, our results suggested that BCE alleviated menopausal lipid metabolism abnormality. TG, TC, and LDL-C are risk factors of dyslipidemia, arteriosclerosis, and cardiovascular disease. Moreover, it is known that serum glucose level rises due to a decrease in estrogen levels, causing diabetes and insulin resistance in menopausal women and OVX animals [33,34]. In this study, the serum glucose levels in OVX Ctrl rats were higher than those of sham rats, but BCE intake slightly decreased them; however, there was no significant difference between them (Supplementary Table S1).

### 3.4. Serum Leptin and Adiponectin Levels

Adipocytokine is a general term for cytokines, such as adiponectin and leptin, secreted from adipose tissues. Levels of these adipocytokines increased in OVX rats more than in sham rats, but decreased with BCE intake (Table 2). Adiponectin can prevent arteriosclerosis, enhance the action of insulin, and lower blood pressure, while leptin reduces the appetite; these adipocytokines are effective in treating lifestyle-related diseases [35,36]. Adiponectin and leptin concentrations increase due to late postmenopause in women and estrogen deficiency in animals, such as OVX mice [37,38,39]. The results of this study were consistent with these previous reports, suggesting that this is a compensatory effect due to weight gain and adipocyte growth.

### 3.5. Evaluation of Hepatic Steatosis and Inflammation

**Figure 3:**
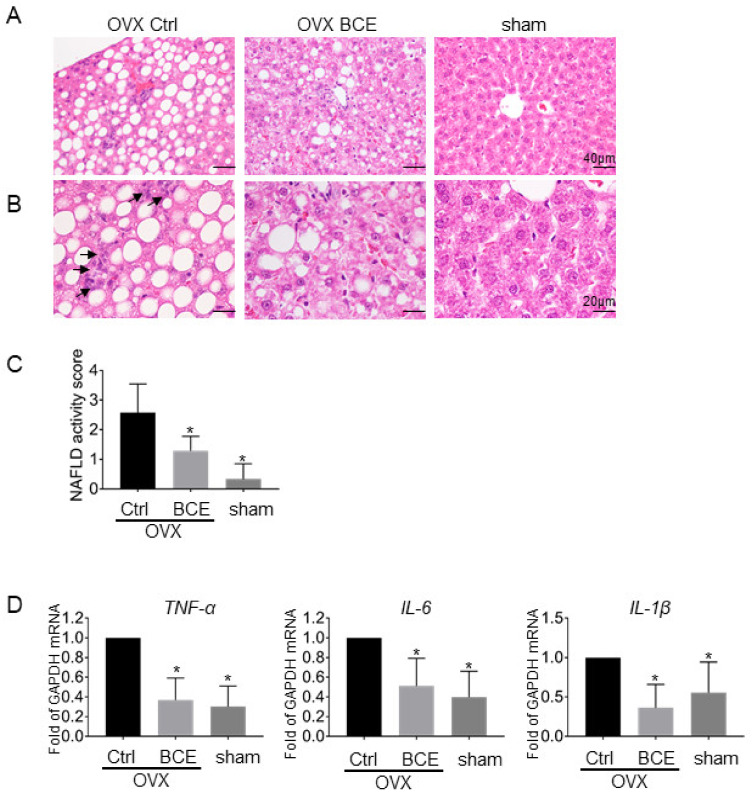
Effects of BCE on liver steatosis. Images of paraffin-embedded hematoxylin and eosin-stained liver sections of OVX rats treated with 3% BCE for 3 months (OVX BCE, n = 9), OVX rats without BCE treatment (OVX Ctrl, n = 10), and sham surgery rats without BCE treatment (sham, n = 9). Magnifications of the upper and lower images are (**A**) 200× and (**C**) 400×, respectively, and scale bars are 40 μm and 20 μm, respectively. (**B**) Nonalcoholic fatty liver disease (NAFLD) activity scores in the liver were estimated in each of the three fields. Data represent the means ± SD. * *p* < 0.05 vs. Ctrl. (**D**) Effects of BCE on mRNA levels of liver inflammatory marker genes. Total mRNA levels in liver tissues from rats of each treatment group were quantified by RT-qPCR. Relative expression of *TNF-α*, *IL-6*, and *IL-1β* was normalized with that of *GAPDH*. Data represent the means ± SD of the means from three rats. * *p* < 0.05 vs. Ctrl.

Menopausal women and OVX animals may develop NAFLD from dyslipidemia. In this study, we examined whether BCE was effective in preventing NAFLD onset by analyzing the liver of OVX rats. Hematoxylin and eosin staining revealed no steatosis in sham rats, but marked steatosis in OVX Ctrl rats; ingestion of BCE decreased the degree of steatosis (Figure 3A). Inflammatory foci and balloons in the liver were not observed, but mild inflammation such as lymphocyte infiltration was detected in OVX Ctrl rats (Figure 3B, black arrow). In contrast, no inflammation was observed in the OVX BCE or sham groups. The NAFLD activity score in the sham group was 0.3 ± 0.5, and it increased to 2.6 ± 0.9 (*p* = 0.0016) in the OVX Ctrl group. However, it decreased to 1.3 ± 0.5 (*p* = 0.006, Figure 3C) in the OVX BCE group. Furthermore, in the livers of OVX Ctrl rats, the expression of hepatic inflammatory marker genes such as *TNF-α*, *IL-6*, and *IL-1β* was higher than that in sham rats, but their levels decreased after BCE intake (Figure 3D).

Feeding OVX mice with a high-fat diet causes liver damage, indicated by an increase in the level of liver damage markers such as serum AST and ALT [30]. In this study, the serum AST and ALT levels increased in the OVX Ctrl rats compared to sham rats and decreased after BCE intake; however, the differences were not significant. Furthermore, there was no change in the serum level of the liver damage marker γ-GT (Supplementary Table S1).

Fat accumulation is known to cause liver fibrosis and weight increase, and blackcurrant was effective in preventing obesity-induced NASH caused by high-fat diet consumption in a previous study [21]. However, severe fibrosis progression (Figure 3A,C) and an increase in liver weight (Supplementary Figure S1) in the OVX Ctrl rats was not observed in this study. Only mild liver inflammation was observed because no high-fat diet was fed. However, BCE intake alleviated liver steatosis progression and the expression of inflammatory genes such as *TNF-α*, *IL-6*, and *IL-1β* in the OVX rats, speculating that daily intake of BCE was effective in preventing NAFLD and NASH in this non-high-fat diet menopausal model rat.

This study used OVX rats as a menopausal dyslipidemia model, and BCE reduced this dyslipidemia. Since we have previously found that BCE has an effect of phytoestrogens [15,16,18,19,20], it is speculated that BCE may alleviate menopausal dyslipidemia via estrogen signaling. On the other hand, the ingestion of a high-fat food promotes oxidative stress [40,41] and induces dyslipidemia, and it is known that anthocyanins [42,43] and polyphenols [44] have antioxidant potentials and have an effect of reducing dyslipidemia. Polyphenols, such as flavanone [45] and resveratrol [46], as activators of the nuclear receptor, peroxisome proliferator-activated receptor γ, are known to promote adipocyte differentiation. Anthocyanins are also known to bind to some nuclear receptors [47]. Therefore, BCE may function in this menopausal dyslipidemia in addition to its effect as a phytoestrogen, and it is necessary to study this further.

## 4. Conclusions

So far, it has been known that BCE has a phytoestrogen effect, but it is unknown whether it has an effect on menopausal lipid abnormalities. In this study, increased body weights, fat weights, and adipocyte diameters in OVX rats were reduced by the ingestion of BCE. In addition, serum lipids, such as triglyceride and cholesterol, were also reduced. Furthermore, hepatic steatosis and levels of *TNF-α*, *IL-6*, *IL-1β* inflammatory genes increased in OVX rats, but were reduced after BCE intake. This is the first report to show that BCE intake is effective in preventing lipid metabolism abnormality and liver steatosis in menopausal model rats. The results of this study suggest that daily BCE intake is effective in preventing lipid metabolism abnormalities in rats with low estrogen secretion; however, these results should be confirmed in studies with menopausal women to warrant its future use in clinical settings.

## Figures and Tables

**Table 1:** Serum lipid profile in OVX Ctrl (OVX Ctrl) and sham (sham) rats fed with regular diet and OVX rats treated with BCE diet (OVX BCE) after 3 months.

	OVX Ctrl	OVX BCE	Sham
TG (mg/dL)	269.8 ± 57	151.4 ± 64.6 *	212.3 ± 40.2 *
TC (mg/dL)	213.4 ± 98.8	140.5 ± 12.6 *	98.2 ± 53.3 *
LDL-C (mg/dL)	43 ± 5.8	31.7 ± 7.7 *	26.2 ± 14.8 *
HDL-C (mg/dL)	66.2 ± 28	53.1 ± 26.9	65.7 ± 15.5

Data represent the means ± SD of 9–10 animals. * *p* < 0.05 vs. Ctrl.

**Table 2:** Serum adipocytokine levels in OVX Ctrl (OVX Ctrl) and sham (sham) rats, and OVX rats treated with BCE diet (OVX BCE) after 3 months.

	OVX Ctrl	OVX BCE	sham
Adiponectin (μg/mL)	21.2 ± 4.3	16.1 ± 3.4 **	12.0 ± 4.9 **
Leptin (ng/mL)	1.96 ± 0.56	1.29 ± 0.37 *	1.23 ± 0.41 *

Data represent the means ± SD of 9–10 animals. * *p* < 0.05 and ** *p* < 0.01 vs. Ctrl.

## Supplementary Materials

The following are available online at https://www.mdpi.com/2072-6643/12/5/1541/s1, Table S1: Serum glucose and liver injury marker levels of OVX Ctrl and sham (sham) rats fed with regular diet and OVX rats treated with BCE diet (OVX BCE) for 3 months. Figure S1: Effect of BCE on rat liver weight.
