# Supplementary material for: Blackcurrant (Ribes nigrum) Extract Prevents Dyslipidemia and Hepatic Steatosis in Ovariectomized Rats
Source: Nutrients. 2020 May 25;12(5):1541. doi: 10.3390/nu12051541 (PMC7284623; doi:10.3390/nu12051541)
Supplement: Supplementary file 1 [file nutrients-12-01541-s001.pdf]

Supplementary Table S1. Serum glucose and liver injury marker levels of OVX Ctrl and sham (sham) groups fed with regular diet and OVX rats treated with BCE diet (OVX BCE) for 3 months.

	**OVX Ctrl**	**OVX BCE**	**sham**
Glucose (mg/dL)	173.4 ± 55.9	165 ± 48.6	140.6 ± 37.9
AST (U/L)	55 ± 28	36 ± 14	29 ± 16
ALT (U/L)	18 ± 8	14 ± 5	14 ± 5
γ-GT (U/L)	13 ± 7	14 ± 11	12 ± 7

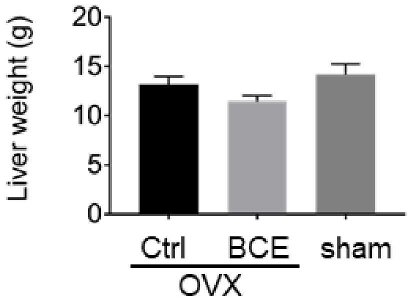

Supplementary Figure S1. Effect of BCE on rat liver weight. OVX rats treated with 3% BCE for 3 months (OVX BCE, n=9), OVX rats without BCE treatment (OVX Ctrl, n=10), and sham surgery rats without BCE treatment (sham, n=9).
